# Supplementary figures and images for: Integrating multiple microarray dataset analysis and machine learning methods to reveal the key genes and regulatory mechanisms underlying human intervertebral disc degeneration
Source: PeerJ. 2020 Oct 13;8:e10120. doi: 10.7717/peerj.10120 (PMC7566771; doi:10.7717/peerj.10120)

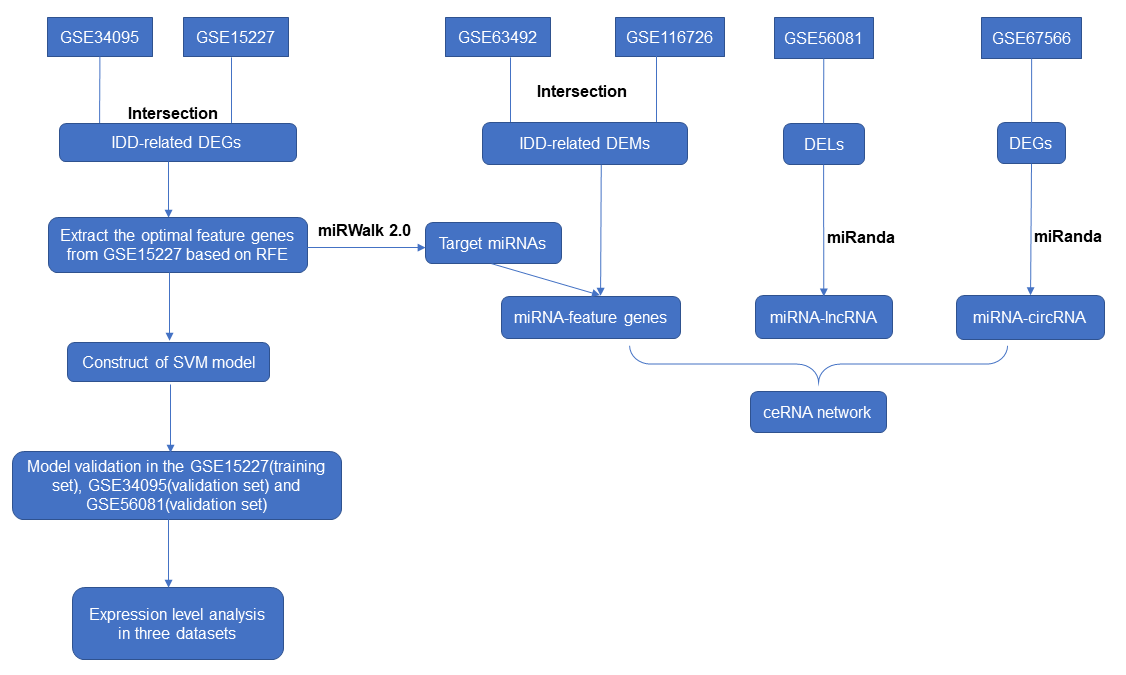

Supplement: Supplemental Information 1 — IDD, intervertebral disc degeneration; DEG, differential expression gene; RFE, recursive feature elimination; SVM, support vector machine; DEM, differential expression miRNA; DEL, differential expression lncRNA; DEC, differential expression circRNA; ceRNA, competing endogenous RNA. [file peerj-08-10120-s001.png]

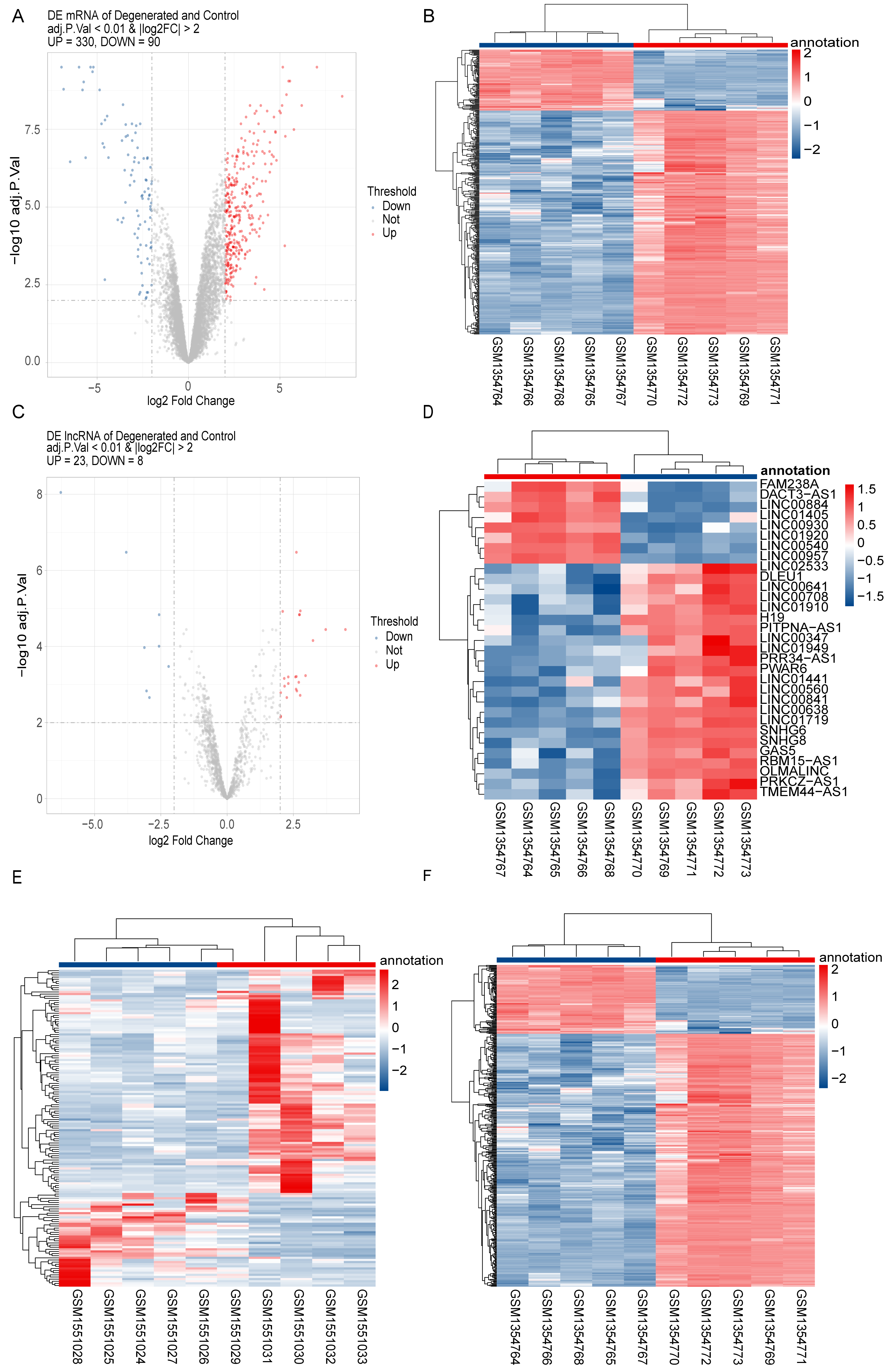

Supplement: Supplemental Information 2 — Volcano plots represent the DEGs (A) and DELs (C) of the degenerative disc samples and control disc samples, respectively, in the GSE56081 dataset. Hierarchical cluster heatmaps represent the DEGs (B) and DELs (D) of the degenerative disc samples and control disc samples, respectively, in the GSE56081 dataset. Hierarchical cluster heatmaps represent the DEMs in the GSE63492 dataset (E) and the DECs (F) in the GSE67566 dataset. Blue represents the downregulated and red represents the upregulated. [file peerj-08-10120-s002.png]
